# IL-6 and Leptin Are Potential Biomarkers for Osteoporotic Fracture Risk Assessment and Prediction of Postmenopausal Women with Low Bone Mass: A Follow-Up Study Using a Regional Sample Cohort

**DOI:** 10.1155/2022/8691830

**Published:** 2022-08-10

**Authors:** Xu Wang, Yili Zhang, Baoyu Qi, Kai Sun, Chuanrui Sun, Ning Liu, Shengjie Fang, Xu Wei, Yanming Xie, Liguo Zhu

**Affiliations:** ^1^Wangjing Hospital, China Academy of Chinese Medical Sciences, Beijing, China; ^2^School of Traditional Chinese Medicine & School of Integrated Chinese and Western Medicine, Nanjing University of Chinese Medicine, Nanjing, China; ^3^Institute of Basic Research in Clinical Medicine, China Academy of Chinese Medical Sciences, Beijing, China

## Abstract

Osteoporotic fracture, a major complication which is known as the outcome postmenopausal osteoporosis, seriously threatens the health of postmenopausal women. At present, the traditional osteoporotic fracture prediction methods are characterized by inconvenient application and time-consuming statistical results, while predictive serum biomarkers can make up for this shortcoming. Accurate and advanced risk prediction of osteoporotic fracture is meaningful to early prevention and intervention, effectively avoiding the risk of this disease and the secondary fracture in the surgical treatment. In this study, based on the BEYOND cohort, a 2-year follow-up study was conducted after subjects participated to survey if OF occurred. Independent sample *t-*test and Mann–Whitney *U*-test were used to analyze the differences of bone metabolism biomarkers between the OF and non-OF group. Cox proportional hazard model was used to screen the potential biomarkers might be used to predict OF risk. ROC curves and AUCs were used to analyze the predictive accuracy, and the Delong's test was used to compare the differences between the AUCs. 15 postmenopausal women with low bone mass and OF were found, and other 60 subjects without OF were matched with 1 : 4, age, and BMI classification as control group. The serum IL-6 (OR = 1.139, 95%CI = 1.058 − 1.226) and leptin (OR = 0.921, 95%CI = 0.848 − 1.000) were found as OF risk predictive biomarkers for postmenopausal women with low bone mass with high accuracy (IL − 6 = 0.871) (leptin = 0.813) and accuracy enhanced when they were combined (AUC = 0.898). The results of Delong's test showed that the difference of AUC between leptin and IL-6&Leptin was meaningful (*P* = 0.024) but meaningless between IL-6 and leptin (*P* = 0.436), IL-6 and IL-6&Leptin (*P* = 0.606). To sum up, IL-6 and leptin are the predictive biomarkers of OF for postmenopausal women with low bone mass. The IL-6 can improve the prediction accuracy of leptin (*P* = 0.024), but not vice versa (*P* = 0.606). *Trial Information*. Registered on the Chinese Clinical Trial Registry already. (Registration Number: ChiCTR-SOC-17013090).

## 1. Introduction

Postmenopausal osteoporosis (PMOP) [1], a serious public health problem, is the major type of osteoporosis (OP) characterized by bone tissue microstructure damaged and bone fragility increased, resulting in a higher fracture risk [2] for postmenopausal women. The prevalence of this condition has been reported to be 29.0% in women over 50, equating to 49 million people in China [3], which is one of the most prevalent metabolic bone diseases of Chinese elderly women. OF, being the final outcome and the most serious complication of PMOP, seriously threatens the health of postmenopausal women. Due to many difficulties in the treatment of the disease such as high risk of secondary fractures [4], it is necessary to accurately predict the risk of OF and prevent it in advance. Early prediction of the disease prior to the occurrence of OF followed by effective prevention of appropriate treatment can reduce fracture risk.

Currently, dual-energy X-ray absorptiometry (DEXA) [5] is the normal approach of obtaining bone mineral density (BMD) to assess the risk of OF. Meanwhile, some established risk factors like *T*-scores [6] and prior fracture [7] also took part in predicting imminent risk of fracture, as falls [8], physical functioning [9], lifestyle [10, 11], and general health [12]. Nevertheless, the radiation, high cost of imaging equipment and the time-consuming, recall bias of questionnaire survey cannot be ignored when these methods are used to collect the prediction information. A faster and more efficient predictive method that is easier to screen on a large scale should be found, which is beneficial to the prevention of this high-risk disease. Serum biomarkers detection is an effective way to solve the abovementioned urgent problems.

At present, various pathogenesis of POMP and OF has been proposed, such as oxidative stress [13], inflammatory response [14], lipid metabolism [15], and angiogenesis [16]. Meanwhile, many relevant biomarkers have shown the correlation between osteoporosis and already been used to auxiliary diagnose OP and assess bone metabolism, such as leptin, Interleukin-6 (IL-6), insulin-like growth factor 1 (IGF-1), and vascular endothelial growth factor (VEGF) [17–19]. However, whether these biomarkers could be used to predict the risk of OF have no explicit evidence. Therefore, based on the BEYOND cohort [20] conducted by our team from 2017 to 2018 and its follow-up work on October, 2019, 15 patients with OF and 60 participants with low bone mass matched by age and BMI classification were selected to screen for predictive serum biomarkers of OF.

## 2. Materials and Methods

In this study, based on a cross-sectional and prospective follow-up study, we used Cox proportional hazards model to examine the relationships between the serum biomarkers and risk for OF among postmenopausal women with low bone mass. We focused on a 2-year period to evaluate the OF risk in this study.

The Ethics Committee of Wangjing Hospital of China Academy of Chinese Medical Sciences (approval number: WJEC-KT-2017-020-P001) approved this research.

This study had been registered on the Chinese Clinical Trial Registry already. (website: http://www.chictr.org.cn) (Registration Number: ChiCTR-SOC-17013090).

### 2.1. Data Source

This study used data from the study of BEYOND cohort, a cross-sectional and prospective study established from December 2017 to July 2018. It included 1540 participants from 10 communities in Chaoyang District and Fengtai District of Beijing as prospective data that had served as the basis for studies of OP and OF. Participants accepted examinations and questionnaire survey when they joined in this cohort and they were accepted a telephone interview on October 2019. Data on BMD, serum biomarkers, body mass index (BMI), lifestyle, medical history, medication use, and physical function were collected at the first examination visit. Participants reported on fractures and time, causes, locations, and treatments during the follow-up study.

Among 1,540 participants of BEYOND cohort, we included postmenopausal women with low bone mass (including osteopenia and osteoporosis) who were not diagnosed with diabetes, thyroid disease, kidney disease, and rheumatoid disease (disease cause secondary OP) as our follow-up interviewees (*n* = 712). Among these individuals, we selected participants suffered OF in these two years (*n* = 15) as OF group.

**Figure 1:**
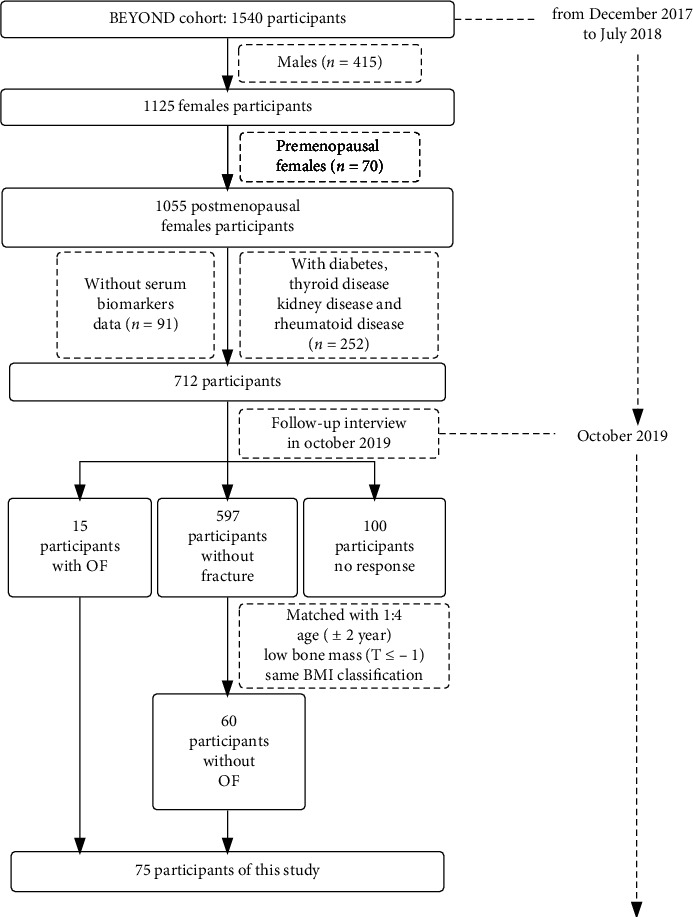
Participants screening process.

Then, the OF group was matched 1 : 4 with participants in the 712 who had no suffered OF in these two years. The non-OF group was selected from the postmenopausal women with low bone mass who were interviewed. Matching was performed based on their age (±2 year) and BMI classification (low weight: BMI < 18.5, normal: 18.5 ≤ BMI < 24, overweight: 24 ≤ BMI < 28, obesity: BMI ≥ 28). Finally, 1 : 4 matching resulted in the inclusion of 15 OF patients and 60 non-OF participants (Figure 1).

### 2.2. Diagnostic Criteria

Dual-energy X-ray absorptiometry device (Hologic, WI, USA) was used to assess the value of BMD (g/cm^2^). The diagnosis of OP was based on the criteria outlined by the WHO and Chinese guidelines [21], *T* value > -1.0 was common; −2.5 ≤ T value ≤ −1.0 was osteopenia; *T* value < -2.5 was osteoporosis.

### 2.3. Study Outcomes

The primary outcome was OF. According to the definition of OF in Primary Osteoporosis Diagnosis and Treatment Guidelines published by Chinese Journal of Osteoporosis and Bone Mineral Research in 2017 [22], low bone mass (*T* ≤ −1.0) combined with low energy fractures of the proximal humerus, pelvis, and distal forearm all belong to OF. Therefore, low-energy fractures in patients with osteoporosis and osteopenia in the BEYOND cohort will be included in this study.

### 2.4. Biomarker Detection

Fasting blood samples of the participants were collected between 8 a.m. and 9 a.m. in sitting position. The measurements were conducted through automated electrochemiluminescence immunoassay system (Roche, Cobas E601, Germany). In addition, the serum segregated for detection was stored at -80°C. Detected biomarkers include serum calcium, serum phosphorus, serum magnesium, 25-hydroxy-vitamin D [25(OH)VitD_3_], *β* isomer of C-terminal telopeptide of type I collagen (*β*-CTx), osteocalcin (OST), parathyroid hormone (PTH), alkaline phosphatase (ALP), type I procollagen amino-terminal peptide (P1NP), IGF-1, IL-6, leptin, and VEGF.

### 2.5. Statistical Analysis and Accuracy Assessment

Kolmogorov-Smirnov test was used to test continuous variables for a normal distribution and was presented as the median with an interquartile range. *t*-test or Mann–Whitney *U*-test was used to analyze continuous detection indexes and the variables which *P* < 0.2 were included in the model [23]. Furtherly, the Cox proportional hazards model was used to explore the predictive biomarkers associated with OF. Based on the principle of modeling and variable selection, ROC curve and area under curve (AUC) were used to verify the accuracy of the model. *P* < 0.05 indicated that the difference between the two groups was statistically significant.

All statistical analysis was carried out with SPSS Statistics 22.0 software. ROC curves were obtained by Medclac 21.0 software. Figures were created in GraphPad Prism 8.0 (GraphPad Software, CA, USA).

## 3. Results

### 3.1. Study Population and Participant Characteristics

15 OF patients and 60 non-OF participants were selected in this study. Among the 15 patients with OF, 7 participants were osteopenia, and the others were osteoporosis; 14 were due to fall, and one person was due to cough. There were 8 limb fractures, 5 spine fractures, and 2 hip fracture (Table 1). The oldest participant was 74 years old, the youngest was 51, median age of 15 patients was 61.00 (55.00, 70.00) years. Among the 15 patients, 5 were of normal weight, 6 were overweight, and 4 were obesity.

The median years of non-OF group were 61.50 (55.00, 69.75) years, the oldest was 75 years old, and the youngest was 50 years old. Meanwhile, their BMI classification was consistent with 15 patients completely.

### 3.2. Normality Test

According to the results of Kolmogorov-Smirnov and Shapiro-Wilk test, the ages, menopausal age, pregnancies times, delivery times, serum 25(OH)VitD3, *β*-CTx, ALP, P1NP, IL-6, and VEGF did not obey the normal distribution; the serum phosphorus, serum calcium, serum magnesium, OST, PTH, IGF-1, and leptin obeyed the normal distribution. The results of the normality test were shown in Table 2. Mann–Whitney *U* test was used to analysis the date did not obey the normal distribution, and *t*-test was used to analysis the date obey the normal distribution.

### 3.3. Univariate Analysis of Population Information and Biomarkers

Mann–Whitney *U* test was used to analyze the nonnormally distributed data, and *t*-test was used to analyze the normally distributed data. According to the results of univariate analysis, the differences of age, menopause age, and delivery times between the two groups have no statistically significant. Meanwhile, the differences of serum IGF-1(*P* = 0.004), IL-6 (*P* < 0.001), and leptin (*P* < 0.001) between the OF group and the non-OF group are statistically significant (Table 3). In addition, six markers are found that the *P* value was less than 0.2, including serum calcium, OST, IGF-1, IL-6, leptin, and VEGF.

### 3.4. OF Risk Factor Analysis

Cox proportional hazard model was established to find the biomarkers could be used to predict OF. The variables whose *P* value was less than 0.2 (serum calcium, OST, IGF-1, IL-6, leptin, and VEGF) were included in the Cox proportional hazards model, and the time was set as the number of month from they joined the cohort to follow-up study visited. The results showed that the differences between IL-6 and leptin were statistically significant. IL-6 was predictive risk biomarkers for OF in this population (*P* < 0.001, OR = 1.139), while leptin was a protective factor for OF (*P* < 0.049, OR = 0.921) (Table 4).

### 3.5. Serum IL-6, Leptin between OF and Non-OF Group

**Figure 2:**
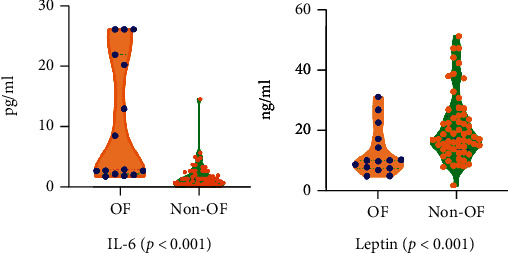
The comparison of IL-6 and leptin between OF and non-OF group. The blue points are the data of OF group participants, and the orange points are the data of non-OF group participants.

According to the results of Cox proportional hazards model, IL-6 and leptin were found as a OF risk prediction model. All serum concentration of these biomarkers is shown in Figure 2. We could find that the serum IL-6 level of OF group was significantly higher than that in the non-OF group (*P* < 0.001), while the result of leptin was opposite (*P* < 0.001).

### 3.6. ROC Curve and AUC Analysis

**Figure 3:**
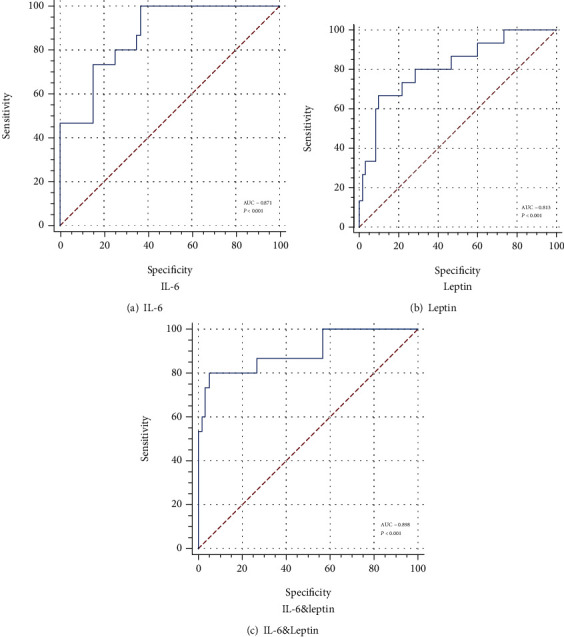
ROC curves of IL-6 and leptin. (a) ROC curve of IL-6 (AUC = 0.871, *P* < 0.001). (b) ROC curve of leptin (AUC = 0.813, *P* < 0.001). (c) ROC curve of IL-6&Leptin (AUC = 0.898, *P* < 0.001).

The ROC curves showed an accurate discrimination performance for these two biomarkers. Herein, we showed the OF prediction accuracy of IL-6 (Figure 3(a)), leptin (Figure 3(b)), and IL-6&Leptin (Figure 3(c)). The AUCs for different types were shown in Figure 3 and Table 5. The AUC value of IL-6 (AUC = 0.871) was close to leptin (AUC = 0.813). And the OF predictive accuracy increased when they were applied together (AUC = 0.898).

Furthermore, the Delong's test was used to analyze the differences of AUCs between every biomarkers group. We found that the difference of AUC between leptin and IL-6&Leptin was meaningful (*P* = 0.024), which showed the OF prediction accuracy of IL-6&Leptin group was higher than leptin. However, the difference of AUC between IL-6 and leptin (*P* = 0.436) and IL-6 and IL-6&Leptin (*P* = 0.606) was all meaningless (Table 6).

## 4. Discussion

OF is the most serious complication of postmenopausal osteoporosis [24] and active prevention of it can be beneficial to prolong life expectancy and improve quality of life for the elderly [25]. Based on the characteristics of OF, early prevention is particularly more important than treatment [26]. In this study, based on the information and data from follow-up study and the results of Cox proportional hazards model, we focused on the predictive effect of IL-6 and leptin. These two biomarkers are associated with inflammatory response, oxidative stress, lipid metabolism, and bone tissue formation and destruction.

Inflammatory microenvironment mediated by the immune system in vivo is considered to be a major reason of abnormal bone metabolism. It may lead to osteoclast activation to accelerate bone loss [27, 28], increase the risk of fracture [29, 30], and slow down the healing rate of fracture [31, 32]. Many medicines are used to treat osteoporosis and repair the bone tissue by alleviating the expression of tissue inflammatory factors [33–35]. Meanwhile, inflammatory factors infiltration caused by many inflammatory diseases can also lead to abnormal bone metabolism, resulting in increased fracture probability, such as chronic pancreatitis [36, 37], chronic enteritis [38], hepatitis [39], and chronic obstructive pulmonary disease [40]. IL-6 is a common inflammatory factor whose effect on bone metabolism has been confirmed already [41]. A randomized controlled clinical trial led by Saribal et al. [42] included 40 patients with hip fractures due to osteoporosis and 40 age-matched nonosteoporotic healthy controls and found that the difference of IL-6 levels between this two group was statistically significant, which considered the relevance of IL-6 and OF. In 2014, based on a cohort with a total of 9704 Caucasian women, Barbour et al. [43] found that women in the highest quartile of IL-6 had a significantly higher risk of hip fractures compared to women in the lowest quartiles, which suggested that IL-6 can predict fractures of women. However, the association between IL-6 and OF still needs more evidence.

Leptin is a hormone with multiple functions that can act locally and systemically. It not only involved in lipid metabolism but also associated with inflammatory response [44] and oxidative stress [45]. In recent years, the relationship between leptin and bone metabolism has been confirmed gradually. Leptin stimulates the differentiation of stromal cells to osteoblasts [46], increases proliferation of osteoblasts [47], and inhibits osteoclastogenesis. Deficiency in leptin signaling, through knockout of the Leptin receptor gene, decreases bone volume and BMD [48], indicating the important role of leptin in bone homeostasis. However, there is no consensus on whether leptin can be used to predict OF. Based on a cohort of 1167 postmenopausal women and a 25-year follow-up interview, an epidemiological survey [49] conducted in Japan showed that leptin levels and postmenopausal women were significantly independent risk factors for long bone fractures and vertebral fractures. However, another research [50] found that there was no significant difference in serum leptin between osteoporosis patients and nonosteoporosis patients. In fact, the effect of leptin on bone metabolism is related to factors such as weight and obesity [51]. Some scholars believe that the high expression of leptin in obese patients (BMI > 28) may have a lower effect on bone metabolism than those with normal weight [52]. Therefore, when using leptin to predict fracture risk, it cannot be discussed separately from weight or obesity. In this study, we match subjects by same BMI classification, so it can be considered that the relationship between fracture and leptin is reliable.

In addition, IL-6 and leptin interact with each other too. Both these two biomarkers can be produced by adipose tissue [53] and mediated many pathological reaction together. Hoffmann et al. [54] found that leptin administration within the subphysiological to physiological range diminished circulating proinflammatory IL-6 in female mice and reduction of IL-6 gene expression in adipose tissue, as well as decreased adipose tissue macrophage infiltration might contribute. However, Wueest and Konrad [55] found that IL-6 could induce the release of leptin from adipocytes, which was contrary to the conclusion of the previous article. In fact, there are few studies on the relationship between IL-6, leptin, and OF, but it cannot be ignored.

Meanwhile, according to the results of Delong's test, IL-6 can improve the prediction accuracy of leptin (*P* = 0.024), but not vice versa. It can be considered that the prediction accuracy of IL-6&Leptin is not better than IL-6, and we should use IL-6 to predict OF separately due to economical. However, prediction accuracy of leptin is reliable (AUCs = 0.813), and these two biomarkers have different predictive directionalities to OF, which may have implications for accurate prediction of different populations in the future. Therefore, we preserve the role of leptin in the predictive model.

This study has the following advantages: based on the BEYOND cohort, all 15 patients of OF group suffered this disease after entry into the cohort and before follow-up study, ensuring the reliability of the results. Then, a Cox disease risk prediction method was used to construct a prediction model of OF risk with IL-6 and leptin as the main risk prediction indicators.

This study also has certain limitations. First, only Beijing community subjects were included in our study, which limited the extrapolation of the results of our study. In addition, only two-year follow-up interview had been developed, and the incidence of outcome indicators was low (15/612, 2.45% in 2 years). So as to solve the problem, we matched these 15 patients in a ratio of 1 : 4 with ages and BMI classification. Meanwhile, a latest prevalence study [56] in China found that the clinical fracture prevalence of women over 40 years old in 5 years is 4.3%, as well as the prevalence in 5 years of women in urban is 4.4%. Due to the similar participants and research design, we believe that the OF prevalence of our study is similar to the results of the latest prevalence study in China. Notwithstanding these limitations, the main progress of our study was the follow up interview for a relatively large sample size and established a fracture risk prediction model suitable for the clinical characteristics of Chinese postmenopausal women with low bone mass.

## 5. Conclusion

Overall, evidence based on current findings suggests that serum IL-6 and leptin can be the predictive factors of OF risk for postmenopausal women with low bone mass, but it needs to be proved by long-term follow-up studies with large sample. We will continue to improve the relevant programs and increase the sample size, so as to find higher quality evidences.

## Figures and Tables

**Table 1:** The characteristics of 15 OF patients.

Number	Ages	BMI	Classification	Recruited time	Fracture time	Reason	Location
1	55	26.839	Overweight	June, 2018	August, 2019	Fall down	Ankle
2	52	21.484	Normal weight	June, 2018	December, 2018	Fall down	Ankle
3	58	28.444	Obesity	June, 2018	June, 2019	Fall down	Wrist
4	55	27.142	Overweight	May, 2018	December, 2018	Fall down	Hip
5	74	25.781	Overweight	May, 2018	February, 2019	Fall down	Wrist
6	61	25.631	Overweight	May, 2018	November, 2018	Fall down	Hip
7	58	23.225	Normal weight	May, 2018	September, 2018	Fall down	Lumbar spine
8	66	29.997	Obesity	May, 2018	October, 2018	Fall down	Lumbar spine
9	71	20.937	Normal weight	April, 2018	December, 2018	Fall down	Wrist
10	59	28.134	Obesity	April, 2018	February, 2019	Fall down	Wrist
11	70	25.537	Overweight	April, 2018	September, 2019	Cough	Lumbar spine
12	71	28.228	Obesity	March, 2018	November, 2018	Fall down	Ankle
13	51	21.414	Normal weight	December, 2017	October, 2019	Fall down	Lumbar spine
14	68	23.335	Normal weight	December, 2017	August, 2018	Fall down	Thoracic spine
15	65	27.392	Overweight	December, 2017	October, 2018	Fall down	Elbow

**Table 2:** Normality test of population information and biomarkers.

Group	Kolmogorov-Smirnova			Shapiro-Wilk		
	Statistic	df	Sig.	Statistic	df	Sig.
Ages	0.117	75	0.013	0.947	75	0.003
Menopause age	0.219	75	<0.001	0.890	75	<0.001
Delivery times	0.289	75	<0.001	0.777	75	<0.001
Pausimenia age	0.138	75	0.001	0.949	75	0.004
Serum phosphorus	0.094	75	0.097	0.929	75	<0.001
Serum calcium	0.080	75	0.200^∗^	0.973	75	0.104
Serum magnesium	0.095	75	0.093	0.980	75	0.284^∗^
25(OH)VitD3	0.145	75	0.001	0.898	75	<0.001
*β*-CTx	0.155	75	<0.001	0.936	75	0.001
OST	0.070	75	0.200^∗^	0.975	75	0.136
PTH	0.090	75	0.200^∗^	0.953	75	0.007
ALP	0.124	75	0.006	0.913	75	<0.001
P1NP	0.111	75	0.023	0.962	75	0.024
IGF-1	0.055	75	0.200^∗^	0.994	75	0.987^∗^
IL-6	0.344	75	<0.001	0.500	75	<0.001
Leptin	0.101	75	0.056	0.955	75	<0.001
VEGF	0.243	75	<0.001	0.659	75	<0.001

^∗^ *P* > 0.05, the simple is normally distributed.

**Table 3:** Univariate analysis of population information and biomarkers.

Characteristics	Total (*n* = 75)	OF group (*n* = 15)	Non-OF group (*n* = 60)	*t*/*Z*	*P*
Age	61.00 (55.00, 70.00)	61.00 (55.00, 70.00)	61.50 (55.00, 69.75)	-0.073	0.942
Menopause age	50.00 (48.00, 52.00)	50.00 (48.00, 52.00)	50.00 (47.00, 52.00)	0.481	0.631
Pregnancies times	2.00 (1.00, 3.00)	2.00 (2.00, 3.00)	2.00 (1.00, 3.00)	0.414	0.679
Delivery times	1.00 (1.00, 2.00)	1.00 (2.00, 2.00)	1.00 (1.00, 2.00)	0.903	0.367
Serum phosphorus (mmol/L)	1.47 ± 0.26	1.50 ± 0.24	1.46 ± 0.28	-0.502	0.617
Serum magnesium (mmol/L)	0.95 ± 0.07	0.94 ± 0.05	0.96 ± 0.07	1.067	0.294
Serum calcium (mmol/L)	2.35 ± 0.07	2.38 ± 0.07	2.35 ± 0.07	-1.815	0.074
25(OH)VitD3 (pg/ml)	15.00 (11.50, 17.20)	16.60 (13.40, 18.60)	14.65 (10.85, 17.05)	1.020	0.308
*β*-CTx (ng/ml)	0.29 (0.21, 0.39)	0.30(0.25, 0.34)	0.29 (0.21, 0.39)	-0.020	0.984
OST (ng/ml)	16.14 ± 5.05	15.51 ± 3.52	16.29 ± 5.38	-0.384	0.192
PTH (pmol/L)	3.25 ± 1.07	3.05 ± 1.09	3.30 ± 1.07	-0.874	0.538
ALP (U/L)	83.00 (72.00, 98.00)	83.00 (70.00, 100.00)	83.00 (70.00, 97.75)	-0.388	0.735
P1NP (ng/ml)	56.87 (46.13, 77.17)	62.55 (53.41, 77.94)	56.03 (45.94, 76.87)	0.748	0.454
IGF-1 (ng/ml)	57.46 ± 21.06	73.38 ± 28.34	52.48 ± 16.88	-3.517	0.004^∗^
IL-6 (pg/ml)	1.74 (1.02, 2.70)	2.90 (2.18, 21.74)	0.82 (1.46, 2.18)	4.426	<0.001^∗∗^
Leptin (ng/ml)	22.35 ± 11.35	12.94 ± 7.83	24.71 ± 10.90	3.861	<0.001^∗∗^
VEGF (pg/ml)	123.55 (73.66, 147.72)	137.53 (110.88, 178.20)	119.46 (65.93, 143.46)	1.497	0.134

Data are presented as Mean ± S.D. or Median (*q*_25_, *q*_75_). ^∗^*P* < 0.05, ^∗∗^*P* < 0.001.

**Table 4:** The result of Cox proportional hazards model.

Factor	*β*	SE	Wald	*P*	OR (95% CI)
OST	-0.085	.084	1.024	0.311	0.919 (0.780-1.083)
Serum calcium	10.511	5.551	3.585	0.058	36733.088 (0.691-1951531307.714)
IGF-1	0.009	0.014	0.403	0.525	1.009 (0.981-1.038)
IL-6	0.130	0.038	11.834	0.001^∗^	1.139 (1.058-1.226)
Leptin	-0.082	0.042	3.860	0.049^∗^	0.921 (0.848-1.000)
VEGF	0.000	0.002	0.004	0.950	1.000 (0.996-1.004)

^∗^ *P* < 0.05, ^∗∗^*P* < 0.001.

**Table 5:** Predictive characteristics of IL-6 and leptin.

Biomarkers	Cutoff	Specificity	Sensitivity	AUC	95% CI for AUC
IL-6	0.633	63.33	100.00	0.871	(0.774-0.937)
Leptin	0.567	90.00	66.67	0.813	(0.707-0.894)
IL-6&Leptin	0.750	95.00	80.00	0.898	(0.806-0.956)

**Table 6:** The results of Delong's test.

Biomarkers group	Delong's test	*P*	95% CI
IL-6 vs. leptin	0.779	0.436	-0.0877 - 0.203
IL-6 vs. IL-6&Leptin	0.516	0.606	-0.0747 - 0.128
Leptin vs. IL-6&Leptin	2.260	0.024^∗^	0.0112 - 0.158

^∗^ *P* < 0.05, ^∗∗^*P* < 0.001.

## Data Availability

All data reported in this study are available upon request by contact with the corresponding author.

## Abbreviations

OF: Osteoporotic fracture
AUC: Area under curve
PMOP: Postmenopausal osteoporosis
BMI: Body mass index
OP: Osteoporosis
DEXA: Dual-energy X-ray absorptiometry
BMD: Bone mineral density
IGF-1: Insulin-like growth factor 1
IL-6: Interleukin-6
VEGF: Vascular endothelial growth factor
25(OH)VitD_3_: 25-Hydroxy-vitamin D3
OST: Osteocalcin
PTH: Parathyroid hormone
*β*-CTx: *β* isomer of C-terminal telopeptide of type I collagen
ALP: Alkaline phosphatase
P1NP: Type I procollagen amino-terminal peptide.

## References

[1] Blake J., Cosman F. A., Lewiecki E. M., McClung M. R., Pinkerton J., Shapiro M. (2021). Management of osteoporosis in postmenopausal women: the 2021 position statement of The North American Menopause Society. *Menopause*.

[2] Compston J. E., McClung M. R., Leslie W. D. (2019). Osteoporosis. *Lancet*.

[3] Cheng X., Zhao K., Zha X. (2021). Opportunistic screening using low-dose CT and the prevalence of osteoporosis in China: a nationwide, multicenter study. *Journal of Bone and Mineral Research*.

[4] Li Y. A., Lin C. L., Chang M. C., Liu C. L., Chen T. H., Lai S. C. (2012). Subsequent vertebral fracture after vertebroplasty. *Spine (Phila Pa 1976)*.

[5] Kanis J. A. (2002). Diagnosis of osteoporosis and assessment of fracture risk. *Lancet*.

[6] (1994). Assessment of fracture risk and its application to screening for postmenopausal osteoporosis. Report of a WHO study group. *World Health Organization Technical Report Series*.

[7] Kanis J. A., Johansson H., Harvey N. C. (2021). The effect on subsequent fracture risk of age, sex, and prior fracture site by recency of prior fracture. *Osteoporosis International*.

[8] Nilsson M., Eriksson J., Larsson B., Odén A., Johansson H., Lorentzon M. (2016). Fall risk assessment predicts fall-related injury, hip fracture, and head injury in older adults. *Journal of the American Geriatrics Society*.

[9] Furrer R., van Schoor N. M., de Haan A., Lips P., de Jongh R. T. (2014). Gender-specific associations between physical functioning, bone quality, and fracture risk in older people. *Calcified Tissue International*.

[10] Rozenberg S., Bruyère O., Bergmann P. (2020). How to manage osteoporosis before the age of 50. *Maturitas*.

[11] Zhang L., Liu Q., Zeng X. (2021). Association of dyslipidaemia with osteoporosis in postmenopausal women. *The Journal of International Medical Research*.

[12] Almutlaq N., Neyman A., DiMeglio L. A. (2021). Are diabetes microvascular complications risk factors for fragility fracture?. *Current Opinion in Endocrinology, Diabetes, and Obesity*.

[13] Black D. M., Rosen C. J. (2016). Postmenopausal osteoporosis. *New England Journal of Medicine*.

[14] Bijelic R., Milicevic S., Balaban J. (2017). Risk factors for osteoporosis in postmenopausal women. *Medicinski Arhiv*.

[15] Kimball J. S., Johnson J. P., Carlson D. A. (2021). Oxidative stress and osteoporosis. *The Journal of Bone and Joint Surgery. American Volume*.

[16] Ding W., Xu C., Zhang Y., Chen H. (2020). Advances in the understanding of the role of type-H vessels in the pathogenesis of osteoporosis. *Archives of Osteoporosis*.

[17] Zhang Y., Huang X., Sun K. (2022). The potential role of serum IGF-1 and leptin as biomarkers: towards screening for and diagnosing postmenopausal osteoporosis. *Journal of Inflammation Research*.

[18] Wang T., He C. (2020). TNF-*α* and IL-6: the link between immune and bone system. *Current Drug Targets*.

[19] Watson E. C., Adams R. H. (2018). Biology of bone: the vasculature of the skeletal system. *Cold Spring Harbor Perspectives in Medicine*.

[20] Sun M., Zhang Y., Shen H. (2020). Prevalence of and risk factors for community-based osteoporosis and associated fractures in Beijing: study protocol for a cross-sectional and prospective study. *Front Med (Lausanne).*.

[21] Siris E. S., Adler R., Bilezikian J. (2014). The clinical diagnosis of osteoporosis: a position statement from the National Bone Health Alliance Working Group. *Osteoporosis International*.

[22] Xia W. B., Zhang Z. L., Lin H., Jin X. L., Yu W., Fu Q. (2017). Guidelines for diagnosis and treatment of primary osteoporosis (2017). *Chinese journal of osteoporosis and bone mineral research*.

[23] Greenland S. (1989). Modeling and variable selection in epidemiologic analysis. *American Journal of Public Health*.

[24] Clynes M. A., Harvey N. C., Curtis E. M., Fuggle N. R., Dennison E. M., Cooper C. (2020). The epidemiology of osteoporosis. *British Medical Bulletin*.

[25] Viswanathan M., Reddy S., Berkman N. (2018). Screening to prevent osteoporotic fractures. *Journal of the American Medical Association*.

[26] Eastell R., Rosen C. J., Black D. M., Cheung A. M., Murad M. H., Shoback D. (2019). Pharmacological management of osteoporosis in postmenopausal women: an endocrine society∗ clinical practice guideline. *The Journal of Clinical Endocrinology and Metabolism*.

[27] Yokota K., Sato K., Miyazaki T. (2014). Combination of tumor necrosis factor *α* and interleukin-6 induces mouse osteoclast-like cells with bone resorption activity both in vitro and in vivo. *Arthritis & Rhematology*.

[28] Zhao B., Grimes S. N., Li S., Hu X., Ivashkiv L. B. (2012). TNF-induced osteoclastogenesis and inflammatory bone resorption are inhibited by transcription factor RBP-J. *The Journal of Experimental Medicine*.

[29] Fischer V., Haffner-Luntzer M. (2020). Interaction between bone and immune cells: Implications for postmenopausal osteoporosis. *Seminars in Cell & Developmental Biology*.

[30] Wu D., Cline-Smith A., Shashkova E., Perla A., Katyal A., Aurora R. (2021). T-cell mediated inflammation in postmenopausal osteoporosis. *Frontiers in Immunology*.

[31] Zhou W., Lin Z., Xiong Y. (2021). Dual-targeted nanoplatform regulating the bone immune microenvironment enhances fracture healing. *ACS Applied Materials & Interfaces*.

[32] Feng S. K., Chen T. H., Li H. M. (2021). Deficiency of omentin-1 leads to delayed fracture healing through excessive inflammation and reduced CD31^hi^Emcn^hi^ vessels. *Molecular and Cellular Endocrinology*.

[33] Wang J., He M., Wang G., Fu Q. (2018). Organic gallium treatment improves osteoporotic fracture healing through affecting the OPG/RANKL ratio and expression of serum inflammatory cytokines in ovariectomized rats. *Biological Trace Element Research*.

[34] Vijayan V., Khandelwal M., Manglani K., Gupta S., Surolia A. (2014). Methionine down-regulates TLR4/MyD88/NF-*κ*B signalling in osteoclast precursors to reduce bone loss during osteoporosis. *British Journal of Pharmacology*.

[35] Jo Y. J., Lee H. I., Kim N. (2021). Cinchonine inhibits osteoclast differentiation by regulating TAK1 and AKT, and promotes osteogenesis. *Journal of Cellular Physiology*.

[36] Vujasinovic M., Nezirevic Dobrijevic L., Asplund E. (2021). Low bone mineral density and risk for osteoporotic fractures in patients with chronic pancreatitis. *Nutrients*.

[37] Duggan S. N., Purcell C., Kilbane M. (2015). An association between abnormal bone turnover, systemic inflammation, and osteoporosis in patients with chronic pancreatitis: a case-matched study. *The American Journal of Gastroenterology*.

[38] Bartko J., Reichardt B., Kocijan R., Klaushofer K., Zwerina J., Behanova M. (2020). Inflammatory bowel disease: a nationwide study of hip fracture and mortality risk after hip fracture. *Journal of Crohn's & Colitis*.

[39] Biver E., Calmy A., Rizzoli R. (2017). Bone health in HIV and hepatitis B or C infections. *Ther Adv Musculoskelet Dis*.

[40] Blaschke M., Koepp R., Cortis J. (2018). IL-6, IL-1*β*, and TNF-*α* only in combination influence the osteoporotic phenotype in Crohn’s patients via bone formation and bone resorption. *Advances in Clinical and Experimental Medicine*.

[41] Scheidt-Nave C., Bismar H., Leidig-Bruckner G. (2001). Serum interleukin 6 is a major predictor of bone loss in women specific to the first decade past menopause. *The Journal of Clinical Endocrinology and Metabolism*.

[42] Saribal D., Hocaoglu-Emre F. S., Erdogan S., Bahtiyar N., Caglar Okur S., Mert M. (2019). Inflammatory cytokines IL-6 and TNF-*α* in patients with hip fracture. *Osteoporosis International*.

[43] Barbour K. E., Lui L. Y., Ensrud K. E. (2014). Inflammatory markers and risk of hip fracture in older white women: the study of osteoporotic fractures. *Journal of Bone and Mineral Research*.

[44] La Cava A. (2017). Leptin in inflammation and autoimmunity. *Cytokine*.

[45] Tisato V., Romani A., Tavanti E. (2019). Crosstalk between adipokines and paraoxonase 1: a new potential axis linking oxidative stress and inflammation. *Antioxidants (Basel).*.

[46] Zhang B., Yang L., Zeng Z. (2020). Leptin potentiates BMP9-induced osteogenic differentiation of mesenchymal stem cells through the activation of JAK/STAT signaling. *Stem Cells and Development*.

[47] Holloway W. R., Collier F. M., Aitken C. J. (2002). Leptin inhibits osteoclast generation. *Journal of Bone and Mineral Research*.

[48] Bao D., Ma Y., Zhang X. (2015). Preliminary characterization of a leptin receptor knockout rat created by CRISPR/Cas9 system. *Scientific Reports*.

[49] Nakamura Y., Nakano M., Suzuki T. (2020). Two adipocytokines, leptin and adiponectin, independently predict osteoporotic fracture risk at different bone sites in postmenopausal women. *Bone*.

[50] Mohiti-Ardekani J., Soleymani-Salehabadi H., Owlia M. B., Mohiti A. (2014). Relationships between serum adipocyte hormones (adiponectin, leptin, resistin), bone mineral density and bone metabolic markers in osteoporosis patients. *Journal of Bone and Mineral Metabolism*.

[51] Mpalaris V., Anagnostis P., Anastasilakis A. D., Goulis D. G., Doumas A., Iakovou I. (2016). Serum leptin, adiponectin and ghrelin concentrations in post-menopausal women: is there an association with bone mineral density?. *Maturitas*.

[52] Gkastaris K., Goulis D. G., Potoupnis M., Anastasilakis A. D., Kapetanos G. (2020). Obesity, osteoporosis and bone metabolism. *Journal of Musculoskeletal & Neuronal Interactions*.

[53] Coppack S. W. (2001). Pro-inflammatory cytokines and adipose tissue. *The Proceedings of the Nutrition Society*.

[54] Hoffmann A., Ebert T., Klöting N. (2019). Leptin decreases circulating inflammatory IL-6 and MCP-1 in mice. *BioFactors*.

[55] Wueest S., Konrad D. (2018). The role of adipocyte-specific IL-6-type cytokine signaling in FFA and leptin release. *Adipocytes*.

[56] Wang L., Yu W., Yin X. (2021). Prevalence of osteoporosis and fracture in China: the China osteoporosis prevalence study. *JAMA Network Open*.

